# Protective effects of bioactive phytochemicals from *Mentha piperita* with multiple health potentials

**DOI:** 10.4103/0973-1296.66926

**Published:** 2010

**Authors:** Seyedeh Maryam Sharafi, Iraj Rasooli, Parviz Owlia, Massoud Taghizadeh, Shakiba Darvish Alipoor Astaneh

**Affiliations:** *Department of Biology, Shahed University, Tehran-Qom Express Way, Opposite Imam Khomeini′s shrine, Tehran-3319118651, Iran*; 1 *Medicinal Plants Research Center, Shahed University, Tehran-Qom Express Way, Opposite Imam Khomeini′s shrine, Tehran-3319118651, Iran*; 2 *Faculty of Medicine, Shahed University, P.O.Box 14155-7435, Tehran, Iran*

**Keywords:** Antioxidant, antibacterial, cytotoxicity, cancer, *Mentha piperita*

## Abstract

*Mentha piperita* essential oil was bactericidal in order of *E. coli*> *S. aureus* > *Pseudomonas aeruginosa* > *S. faecalis* > *Klebsiella pneumoniae*. The oil with total phenolics of 89.43 ± 0.58 *µ*g GAE/mg had 63.82 ± 0.05% DPPH inhibition activity with an IC _50_ = 3.9 *µ*g/ml. Lipid peroxidation inhibition was comparable to BHT and BHA. A 127% hike was noted in serum ferric-reducing antioxidant power. There was 38.3% decrease in WBCs count, while platelet count showed increased levels of 214.12%. Significant decrease in uric acid level and cholesterol/HDL and LDL/HDL ratios were recorded. The volatile oil displayed high cytotoxic action toward the human tumor cell line. The results of this study deserve attention with regard to antioxidative and possible anti-neoplastic chemotherapy that form a basis for future research. The essential oil of mint may be exploited as a natural source of bioactive phytopchemicals bearing antimicrobial and antioxidant potentials that could be supplemented for both nutritional purposes and preservation of foods.

## INTRODUCTION

Supplementation of human diet with herbs, containing especially high amounts of compounds capable of deactivating free radicals, besides the fruits and vegetables recommended as optimal sources of antioxidant activity, may have beneficial effects. The benefits resulting from the use of natural products rich in bioactive substances have promoted the growing interest of pharmaceutical, food and cosmetic industries as well as of individual consumers in the quality of herbal products. The oxidative deterioration of lipids is of great concern in the shelf life of foods. Lipid peroxidation leads to development of undesirable off-flavors and decreases the acceptability of foods. In addition, lipid oxidation decreases food safety and nutritional quality by the formation of potentially toxic products and secondary oxidation products during cooking or processing.[1] To prevent and retard lipid oxidation, synthetic antioxidants such as butylated hydroxyanisole (BHA), butylated hydroxytoluene (BHT) and propyl gallate (PG) have been added to lipid-containing foods.[2] However, potential health hazards of synthetic antioxidants in foods, including possible carcinogens, have been reported several times.[3] Antioxidants are compounds that can delay, inhibit, or prevent the oxidation of oxidizable matters by scavenging free radicals and diminishing oxidative stress. Plants contain a wide variety of antioxidant phytochemicals or bioactive molecules, which can neutralize the free radicals and thus retard the progress of many chronic diseases associated with oxidative stress and reactive oxygen species (ROS). The intake of natural antioxidants has been associated with reduced risk of cancer, cardiovascular disease, diabetes and diseases associated with ageing. Studies on dietary free radical scavenging molecules have attracted the attention to characterize phenolic compounds and other naturally occurring phytochemicals as antioxidants.[4] Spices and herbs are generally applied to food which is a nutrient rich environment for most bacteria. The antibacterial activity, however, could be also used in nutrient poor environments, for example, cleaning of food processing devices and depuration of shellfish.[5] A variety of molecules derived from spice possess bioactive properties. Spices are also considered as nutraceuticals in view of their nutritional, medicinal and therapeutical properties. In addition to improving flavor, certain spices and essential oils prolong the storage life of foods by an antimicrobial activity. Spices are primarily used in the food industry for improving the quality of the product. These powdered spices suffer disadvantages, e.g. quality variations from batch to batch caused by uneven distribution of flavor, loss of flavor strength, quality during storage, insect infestation, bacterial contamination, unhygienic nature and inconveniency in bulk handling. To overcome these problems, spice essential oil has come into use in the food industries.[6] Food-borne illness caused by consumption of contaminated foods with pathogenic bacteria and/or their toxins has been of great concern to public health. Controlling pathogenic microorganisms would reduce food-borne outbreaks and assure consumers a continuing safe, wholesome and nutritious food supply.[7,8] The exploration of naturally occurring antimicrobials for food preservation receives increasing attention due to consumer awareness of natural food products and a growing concern of microbial resistance toward conventional preservatives.[9] Bearing in mind the growing use of essential oils, their genotoxicity testing, identification of genotoxic compounds and attempts to improve their safety may be important fields of future research. *Mentha piperita* (Peppermint) is globally and widely used in the forms of oil, extract, leaves, and water. However, very little information is available on physiological, pharmacological and cytotoxic properties of *Mentha piperita* essential oil. Therefore, the present investigation was undertaken to evaluate the protective activity of bioactive phytochemicals from *M. piperita* essential oil.

## MATERIALS AND METHODS

### Equipments and chemicals

The major equipments used were UV-2501PC spectrophotometer, ELISA reader and routine microbiology laboratory equipments. The essential oil was purchased from Zardband company, Tehran, Iran. Microbial and cell culture media and laboratory reagents were from Merck, Germany. Other chemicals were of analytical grade.

### Microbial strain and growth media

*E. coli* (ATCC 25922), *S. aureus* (ATCC 25923), *Streptococcus fecalis* (PTCC 33186), *Pseudomonas aeruginosa* (ATCC 8830) and *Klebsiella pneumoniae* (ATCC 13883) were employed in the study. Bacterial suspensions were made in brain heart infusion (BHI) broth to a concentration of approximately 10 ^8^ cfu/ml. Subsequent dilutions were made from the above suspension, which were then used in the tests.

### Oil sterility test

To ensure sterility of the oils, geometric dilutions ranging from 0.036 to 72.0 mg/ml of the essential oil were prepared in a 96-well microtitre plate, including one growth control (BHI+DMSO) and one sterility control (BHI+DMSO+test oil). Plates were incubated under normal atmospheric conditions, at 37°C for 24 h. The contaminating bacterial growth, if at all, was indicated by the presence of a white ‘“pellet”’ on the well bottom.

### Oil dilution solvent

5 µl of dimethylsulphoxide (DMSO) loaded on sterile blank disks were placed on Mueller Hinton agar plates streaked with suspensions of bacterial strains, and were then incubated at 37°C for 24 hours. There was no antibacterial activity on the plates and hence DMSO was selected as a safe diluting agent for the oil. 10 µl from each oil dilution was added to sterile blank discs. The solvent also served as control.

### Disc diffusion method

The agar disc diffusion method was employed for the determination of antimicrobial activities of the essential oils in question. Briefly, 0.1 ml from 10^8^ CFU/ml bacterial suspension was spread on the Mueller Hinton Agar (MHA) plates. Filter paper discs (6 mm in diameter) were impregnated with 10 µl of the undiluted oil and were placed on the inoculated plates. These plates, after remaining at 4°C for 2h, were incubated at 37°C for 24 h. The diameters of the inhibition zones were measured in millimeters. All tests were performed in triplicate.

### Radical scavenging capacity of the oils

The hydrogen atom or electron donation abilities of the corresponding extracts and some pure compounds were measured from the bleaching of the purple-colored methanol solution of 2,20-diphenylpicrylhydrazyl (DPPH). 50 µl of 1:5 concentrations of the essential oil in methanol was added to 5 ml of a 0.004% methanol solution of DPPH. Trolox (1 mM) (Sigma-Aldrich), a stable antioxidant, was used as a synthetic reference. After a 30 min incubation period at room temperature, the absorbance was read against a blank at 517 nm. Inhibition of free radical by DPPH in percent (I%) was calculated in the following way:

I% = (A_blank_ — A_sample_/A_blank_) × 100;

where A_blank_ is the absorbance of the control reaction (containing all reagents except the test compound), and A_sample_ is the absorbance of the test compound. Tests were carried out in triplicate.

### Lipid peroxidation (LPO) assay

Approximately 10 mg of ²-carotene (type I synthetic, Sigma–Aldrich) was dissolved in 10 ml of chloroform. The carotene-chloroform solution, 0.2 ml, was pipetted into a boiling flask containing 20 mg linoleic acid (Sigma–Aldrich) and 200 mg Tween 40 (Sigma– Aldrich). Chloroform was removed using a rotary evaporator at 40°C for 5 min and to the residue, 50 ml of distilled water was added, slowly with vigorous agitation, to form an emulsion. 5 ml of the emulsion was added to a tube containing 200 µl of essential oils solution and the absorbance was immediately measured at 470 nm against a blank, consisting of an emulsion without β-carotene. The tubes were placed in a water bath at 50°C and the oxidation of the emulsion was monitored spectrophotometrically by measuring absorbance at 470 nm over a 60 min period. Control samples contained 10 µl of water instead of essential oils. Butylated hydroxy anisole (BHA) and butylated hydroxytoluene (BHT), stable antioxidants, were used as synthetic references. The antioxidant activity was expressed as inhibition percentage with reference to the control after 60 minutes incubation using the following equation: AA = 100(DR_C_—DR_S_)/DR_C_,

where AA = antioxidant activity;

DR_C_ = degradation rate of the control = [ln(a/b)/60];

DR_S_ = degradation rate in the presence of the sample = [ln(a/b)/60];

a = absorbance at time 0;

b = absorbance at 60 min.

### Total phenolic content assay

Total phenol content was estimated as gallic acid equivalents (GAE; µg gallic acid/mg extract) as described earlier.[10] In brief, a 100 µl aliquot of dissolved extract was transferred to a 10 ml volumetric flask, containing ca. 6.0 ml H_2_ O, to which was subsequently added 500 µl Folin–Ciocalteu reagent. After 1 min, 1.5 ml 200 g/l Na_2_CO_3_ was added and the volume was made up to 10 ml with H_2_O. After 2 h of incubation at 25°C, the absorbance was measured at 760 nm. Gallic acid (Sigma Co., 0.2-1 mg/ml gallic acid) was used as the standard for the calibration curve, and the total phenolic contents were expressed as mg gallic acid equivalents per gram of tested extracts (Y=0.001*x* +0.0079; r^2^ = 0.997).

### Acute and subchronic toxicity

A 30-day oral toxicity study was conducted in Wistar rats (R*attus norvegicus*; 180–200 g) to determine the potential of C. *cyminum* essential oil to produce toxic effects. The rats of both sexes were housed in temperature-controlled rooms and were given food and water *ad libitum* until used. For the acute toxicity analysis, the essential oil was administered via oral gavage to the rats (*n* = 10 mice per group) at doses ranging from 100 to 2000 mg/kg/day. The results obtained were compared with those for the control animals [3% (v/v) Tween 80 in saline]. The LD_50_ was calculated by the probit method by using SPSS 7.0 for Windows. To investigate the subchronic toxicity of the essential oil, after 30 days of oral administration to rats, the hematological and serum biochemistry parameters were evaluated. Blood samples were collected by puncture in the infraorbital plexus. The blood samples collected on day 0 and day 30 were used for determining red cell and leucocyte counts and for hemoglobin, hematocrit and biochemical parameter analysis. The serum concentrations of urea, creatinine, glutamic-oxalacetic transaminase (GOT) and glutamic-pyruvic transaminase (GPT) and other parameters were determined by using commercial kits. The values obtained were compared within and between the groups.

### Ferric reducing antioxidant power of serum (FRAP)

The antioxidant power of blood serum was determined using FRAP assay.[11] Briefly, 50 µl of the blood serum (normal as well as experimental cells) suspension was added to 1.5 ml of freshly prepared and pre-warmed (37°C) FRAP reagent (300 mM acetate buffer, pH = 3.6, 10 mM TPTZ (tripyridyl-s-triazine) in 40 mM HCl and 20 mM FeCl3.6H2O in the ratio of 10:1:1) and incubated at 37°C for 10 min. The absorbance of the sample was read against reagent blank (1.5 ml FRAP reagent + 50 µl distilled water) at 593 nm. Aqueous solutions of known Fe(II) concentration (FeSO_4_.7H_2_O) were used for calibration of the FRAP assay and antioxidant power was expressed as µg/ml (*y* = 0.0025*x*+0.0005; r^2^ = 0.9976).

### Cytotoxicity assay

The human cervical carcinoma Hela cell line NCBI code No. 115 (ATCC number CCL-2) were obtained from Pasteur Institute, Tehran-Iran. The cells were grown in RPMI 1640 supplemented with 10% fetal calf serum, 1% (w/v) glutamine, 100 U/ml penicillin and 100 µg/ml streptomycin. Cells were cultured in a humidified atmosphere at 37°C in 5% CO_2_. Cytotoxicity was measured using a modified MTT assay. This assay detects the reduction of MTT [3-(4,5-dimethylthiazolyl)-2,5-diphenyltetrazolium bromide] by mitochondrial dehydrogenase, to blue formazan product, which reflects the normal functioning of mitochondrial and cell viability.[12] Briefly, the cells (5 × 10^4^) were seeded in each well containing 100µl of the RPMI medium supplemented with 10% FBS in a 96-well plate. After 24 h of adhesion, a serial of doubling dilution of the essential oil was added to triplicate wells over the range of 1.0–0.005 µl/ml. The final concentration of ethanol in the culture medium was maintained at 0.5% (volume/volume) to avoid toxicity of the solvent.[13] After 2 days, 10 µl of MTT (5 mg/ml stock solution) was added and the plates were incubated for an additional 4 h. The medium was discarded and the formazan blue, which formed in the cells, was dissolved with 100 µl dimethyl sulphoxide (DMSO). The optical density was measured at 490 nm using a microplate ELISA reader. The cell survival curves were calculated from cells incubated in the presence of 0.5% ethanol. Cytotoxicity is expressed as the concentration of drug inhibiting cell growth by 50% (IC_50_), (*y* = 110.12*x*^0.025^; *r*^2^ = 0.9981). All tests and analyses were run in triplicate and mean values were recorded.

### Statistical analysis

All the experimental data are presented as mean ± SEM of three individual samples. Data are presented as percentage of free radical scavenging/inhibition lipid peroxidation on different concentrations of cumin oil. IC_50_ (the concentration required to scavenge 50% of free radicals) value was calculated from the dose-response curves. Antibacterial effect was measured in terms of zone of inhibition to an accuracy of 0.1 mm and the effect was calculated as a mean of triplicate tests. All of the statistical analyses were performed with the level of significant difference between compared data sets being set at *P* < 0.05.

## RESULTS AND DISCUSSION

**Figure 1:**
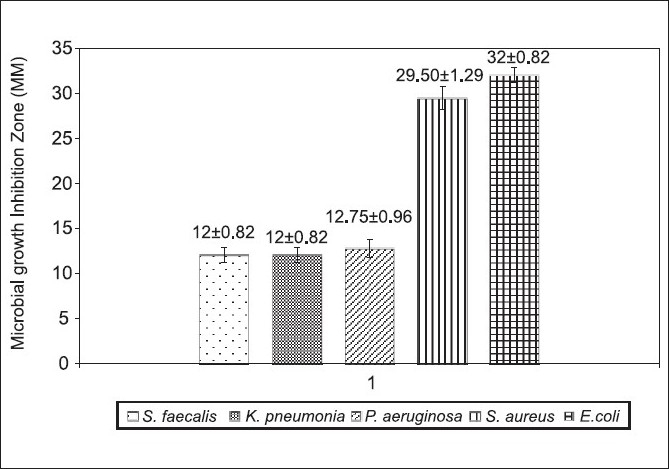
Antimicrobial activity of Mentha piperita essential oil

**Figure 2:**
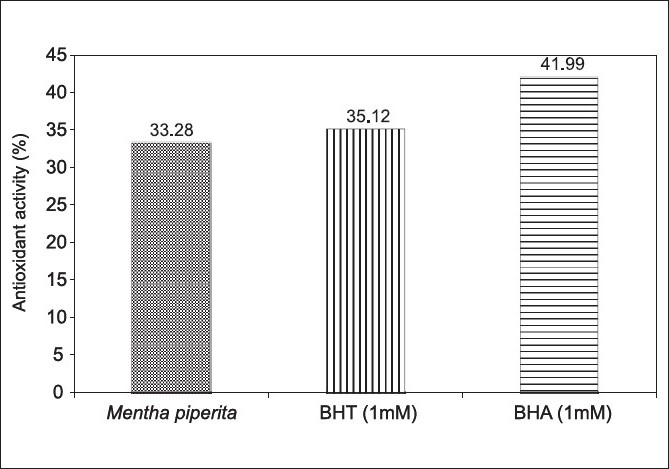
Lipid peroxidation activity of Mentha piperita essential oil

The antibacterial effect of *Mentha piperita* was tested against some bacteria by agar diffusion method. All the test organisms were sensitive to the oil with the sensitivity order of *E.coli*> *S. aureus* > *Pseudomonas aeruginosa*> *S.faecalis* > *Klebsiella pneumoniae* [Figure 1]. The antimicrobial activity of various essential oils including mint (*Mentha piperita*) was evaluated on survival and growth of different strains of *E. coli* O157:H7. The strains of *E. coli* exhibited similar susceptibilities to the action of the essential oils assayed at the inhibition zone diameter range of 16-19mm.[14] Addition of mint essential oil reduced the total viable counts of *S. aureus* about 6–7 logs.[15] Our results seem to be consistent with the above reports. Since essential oils consist of terpenes (phenolics in nature), it would seem reasonable that their mode of action might be related to those of other phenolic compounds.[15] *Mentha* extract (ME) has been reported to have antioxidant and antiperoxidant properties.[16] Hence we attempted to evaluate these properties with the essential oil under study. The oil showed at its maximum 63.82 ± 0.05% inhibition of DPPH activity with an IC_50_ = 3.9 µg/ml [Table 1]. The extracts of the *M. piperita* has shown 93.9 ± 1.68% inhibition of DPPH activity with an IC_50_ = 273 µg/ml.[17] In another study the essential oils of *M. piperita* had an IC_50_ = 2.53 µg/ml in the DPPH assay.[18] Total phenolics of *M. piperita* were 89.43 ± 0.58 µg GAE/mg [Table 1]. Plant phenols and flavonoids are known to inhibit lipid peroxidation by quenching lipid peroxy radicals and reduce or chelate iron in lipoxygenase enzyme and thus prevent initiation of lipid peroxidation reaction.[19] The antioxidant capacities of the essential oil as assessed by different assay methods are summarized in Table 2 and Figure 2. Lipid peroxidation inhibition by *M. Piperita* oil was statistically (*P*>0.05) at the same level of the synthetic antioxidant BHT and lower (*P*<0.001) than BHA [Figure 2]. Many different methods have been established for evaluating the antioxidant capacity of certain biological samples, with such methods being classified, roughly, into one of two categories based upon the nature of the reaction that the method involved.[20] The methods involving an electron-transfer reaction include the total phenolics assay using Folin–Ciocalteu reagent, the TEAC and the DPPH radical-scavenging assay. The radical scavenging effect of *M. piperita* essential oil was found to be 6.6 and 4.17 times more potent than the standard BHT and BHA respectively, but less potent (64%) than Trolox [Table 1]. This suggests that M. piperita essential oil is a good free radical scavenger or hydrogen donor and contributes significantly to the antioxidant capacity of *M. piperita*. The DPPH radical scavenging is a sensitive antioxidant assay and is independent of substrate polarity.[21] DPPH is a stable free radical that can accept an electron or hydrogen radical to become a stable diamagnetic molecule. A significant correlation was shown to exist between the phenolic content and with DPPH scavenging capacity for each spice.[22] Ferric-reducing antioxidant power in the blood sera of the rats gavaged with a daily dose of 100 µl oil showed 127% hike as compared to the control group [Table 2]. The antioxidant activity can be correlated to the moderate phenolic content of the oil. The phenolic content of certain spices appears to correlate well with such spices’ protective effect against peroxynitrite-mediated tyrosine nitration and lipid peroxidation. Such an observation indicates that phenolics present in the spices contributed to such spice-elicited protection against peroxynitrite toxicity.[22] There were some treatment-related effects in hematology parameters [Table 3]. Although the animals gained significant weight but the weight gain was statistically (*P*=0.1) insignificant. There was 38.3% decrease in WBCs count, while platelet count showed increased levels of 214.125% [Table 3]. Clinical chemistry parameters showed significant decrease in uric acid level while total cholesterol and triglycerides levels increased significantly. The interesting observations were of increased good cholesterol (HDL) level that reduced cholesterol/HDL and LDL/HDL ratios to 80% and 45.93% respectively. Thus, *M. piperita* with a high phenolic content and good antioxidant activity can be supplemented for both nutritional purposes and preservation of foods. Recently Dragland *et al*.[23] speculated that the daily intake of 1 g of various potent antioxidant spices makes a relevant contribution to the total intake of antioxidants in a normal diet. Peppermint oil was minimally toxic in acute oral studies. Short-term and subchronic oral studies reported cyst-like lesions in the cerebellum in rats that were given doses of peppermint oil containing pulegone, pulegone alone, or large amounts (>200 mg/kg/day) of menthone. With the limitation that the concentration of pulegone in these ingredients should not exceed 1%, it was concluded that *Mentha piperita* (peppermint) oil, *Mentha piperita* (peppermint) extract, *Mentha piperita* (peppermint) leaves, *Mentha piperita* (peppermint) water are safe as used in cosmetic formulations.[24] At a concentration of 0.02 µl/ml, oil destructed Hela cells by 98.48% [Table 4]. At lower doses, the oil was still toxic to the cells. The volatile oil displayed an excellent cytotoxic action toward the human tumor cell line. The IC_50_ was calculated to be 1×10^-16^ which seem to be indicative of high toxicity of the oil that needs testing with normal healthy cells in order to rule out its hazardous cytotoxicity before it is recommended for use. The oral administration of *Mentha piperita* extract (ME) showed a significant reduction in the number of lung tumors from an incidence of 67.92% in animals given only benzo[*a*]pyrene (BP) to 26.31%. Cancer chemoprevention is defined as the use of chemicals or dietary components to block, inhibit, or reverse the development of cancer in normal or preneoplastic tissue. A large number of potential chemopreventive agents have been identified, and they function by mechanisms directed at all major stages of carcinogenesis.[25] Essential oil constituents have a very different mode of action in bacterial and eukaryotic cells. For bacterial cells they are having strong bactericidal properties, while in eukaryotes they modify apoptosis and differentiation, interfere with the post-translational modification of cellular proteins, induce or inhibit some hepatic detoxifying enzymes. So, essential oils may induce very different effects in prokaryotes and eukaryotes. Peppermint essential oil was reported to be cytotoxic.[26] Thus, peppermint essential oil may be classified as “high toxicity clastogen”,[27] which induces chromosome aberrations by secondary mechanism associated with cytotoxicity. It was suggested[28] that such compounds do not react with DNA and are not genotoxic *in vivo* and usually not carcinogenic. Some reports support the relationship of cytotoxicity with antioxidant activity.[29] Although all *in vitro* experiments hold limitations with regard to possible *in vivo* efficacy, the results of this study deserve attention with regard to antioxidative and possible anti-neoplastic chemotherapy that form a basis for future research. Even though essential oils might not be ideal for the treatment of human cancers, the oil tested certainly deserves some further investigation.

**Table 1:** Total phenolics and mean inhibition of DPPH free radical (%) by Mentha piperita essential oil dilutions

Oil (µg/ml) and synthetic antioxidants	DPPH inhibition (%)	GAE µg Gallic acid/mg sample
10	63.82 ± 0.05	89.43 ± 0.58
5	55.85 ± 0.09	40.43 ± 0.58
2.5	42.77 ± 0.09	15.10 ± 1
0.2	26.50 ± 0.45	9.43 ± 0.58
BHT 1mM	9.63	—
BHA 1mM	15.28	—
Trolox 1mM	99.62	—

**Table 2:** Blood serum Ferric-reducing antioxidant power (FRAP) of Mentha piperita essential oil

Serum FRAP	FeSO4.7H2O equivalent (µg/ml)	Test/control ratio (%)
Control	226.25 ± 5.84	100
*Mentha piperita*	287.4 ± 4.46	127

**Table 3:** Mean hematology and clinical chemistry values of rats blood samples fed with Mentha piperita essential oil

Parameters	Control	Mean value (test)	% change	*P* value
Initial body weight (g)	142.50±2.9	159±3.61	111.58	0.001
Final body weight (g)	157.50±5	198.33±18.93	125.93	0.008
Weight gain (%)	110.53±2	124.95±14.73	114.42	0.106
Erythrocyte count (RBC) (×10^6^/IL)	7.23±1.25	7.90±0.59	109.39	0.430
Total white blood cell (WBC) and differential	9400+668	5800±608	61.7	0.0008
leukocyte count (×10^3^/μl)				
Hemoglobin concentration (HGB) (g/dl)	12.78±1.4	13.53±0.51	105.94	0.434
Hematocrit (HCT) (%)	39.63±1.7	38.5±0.60	97.16	0.338
Platelet count (PLT) (×10^3^/μl)	238500±1658	510666.67±5401.34	214.12	0.0002
Red cell distribution width [RDW (%)]	14.75±2.14	13.97±1.29	94.69	0.602
Mean platelet volume (MPV)	7.600.56	7.07±0.45	92.98	0.236
Mean corpuscular volume (MCV) (fL)	52.93±3.84	50.6±4	95.61	0.471
Mean corpuscular hemoglobin (MCH) (pg)	17.78±1.37	17.33±0.71	97.52	0.638
Mean corpuscular hemoglobin concentration	33.75±1.95	35.47±0.81	105.09	0.217
[MCHC (g/dl)]				
Fasting glucose (GLUC) (mg/dl)	221±7.79	218.33±15.18	98.79	0.77
Blood urea nitrogen (BUN) (mg/dl)	60±5.96	55.23±5.25	92.06	0.322
Blood creatinine (CREA) (mg/dl)	0.64±0.1	0.63±0.10	99.21	0.95
Uric acid	8.18±2.45	4.29±0.85	52.44	0.049
Total cholesterol(CHOL) (mg/dl)	75.75±1	91±1	120.13	0.0000
Triglycerides (TRIG) (mg/dl)	45±9.2	73±8.19	162.22	0.009
HDL	45.50±4	67.30±0.72	147.91	0.0003
LDL	15.05±1.9	10.20±1.71	67.77	0.018
Cholesterol/HDL ratio	1.68±0.16	1.35±0.03	80.71	0.021
LDL/HDL ratio	0.33±0.03	0.15±0.03	45.93	0.0003
SGOT	530.75±68	520.67±69.41	98.10	0.855
SGPT	236.75±9	146.33±16.5	61.81	0.031
Alkaline phosphatase (ALKP) (U/L)	136.75±3	244.67±84.79	178.92	0.063

**Table 4:** Cytotoxicity assay of Mentha piperita essential oil

Oil (µl/ml)	Mean Viable cells	% Death
0.02	1.52 ± 0.5	98.48
0.01	2.17 ± 0.67	97.83
0.0075	2.99 ± 0.75	97.01
0.005	3.89 ± 0.32	96.11
0.0025	5.48 ± 0.84	94.52
0.00125	7.36 ± 0.66	92.64

## CONCLUSION

From the above results, it can be concluded that the tested essential oil exhibited antimicrobial activity against the tested microorganisms and it could be a better natural antioxidant. The essential oil provided comparable antioxidative activity as compared with synthetic antioxidants, which provides a way of screening antioxidants for foods, cosmetics and medicine. Hence, the essential oil of mint may be exploited as a natural source of bioactive phytopchemicals bearing antimicrobial and antioxidant potentials.
